# Derivation and internal validation of the screening to enhance prehospital identification of sepsis (SEPSIS) score in adults on arrival at the emergency department

**DOI:** 10.1186/s13049-019-0642-2

**Published:** 2019-07-16

**Authors:** Michael A. Smyth, Daniel Gallacher, Peter K. Kimani, Mark Ragoo, Matthew Ward, Gavin D. Perkins

**Affiliations:** 1 Clinical Trials Unit, University of Warwick, Coventry, UK; 2 West Midlands Ambulance Service NHS Foundation Trust, Dudley, UK; 3 Midlands Air Ambulance, Dudley, UK; 4 Royal Stoke University Hospital, Stoke on Trent, UK; 5 Heart of England NHS Foundation Trust, Birmingham, UK

**Keywords:** Sepsis, Prehospital, Ambulance, Screening tool, Prediction model

## Abstract

**Background:**

Prehospital recognition of sepsis may inform case management by ambulance clinicians, as well as inform transport decisions. The objective of this study was to develop a prehospital sepsis screening tool for use by ambulance clinicians.

**Methods:**

We derived and validated a sepsis screening tool, utilising univariable logistic regression models to identify predictors for inclusion, and multivariable logistic regression to generate the SEPSIS score. We utilised a retrospective cohort of adult patients transported by ambulance (*n* = 38483) to hospital between 01 July 2013 and 30 June 2014. Records were linked using LinkPlus® software. Successful linkage was achieved in 33289 cases (86%). Eligible patients included adult, non-trauma, non-mental health, non-cardiac arrest cases. Of 33289 linked cases, 22945 cases were eligible. Eligible cases were divided into derivation (*n* = 16063, 70%) and validation (*n* = 6882, 30%) cohorts. The primary outcome measure was high risk of severe illness or death from sepsis, as defined by the National Institute for Health and Care Excellence Sepsis guideline.

**Results:**

‘High risk of severe illness or death from sepsis’ was present in 3.7% of derivation (*n* = 593) and validation (*n* = 254) cohorts. The SEPSIS score comprises the following variables: age, respiratory rate, peripheral oxygen saturations, heart rate, systolic blood pressure, temperature and level of consciousness (*p* < 0.001 for all variables). Area under the curve was 0.87 (95%CI 0.85–0.88) for the derivation cohort, and 0.86 (95%CI 0.84–0.88) for the validation cohort. In an undifferentiated adult medical population, for a SEPSIS score ≥ 5, sensitivity was 0.37 (0.31–0.44), specificity was 0.96 (0.96–0.97), positive predictive value was 0.27 (0.23–0.32), negative predictive value was 0.97 (0.96–0.97), positive likelihood value was 13.5 (9.7–18.73) and the negative likelihood value was 0.83 (0.78–0.88).

**Conclusion:**

This is the first screening tool developed to identify NICE high risk of severe illness or death from sepsis. The SEPSIS score is significantly associated with high risk of severe illness or death from sepsis on arrival at the Emergency Department. It may assist ambulance clinicians to identify those patients with sepsis in need of antibiotic therapy. However, it requires external validation, in clinical practice by ambulance clinicians, in an independent population.

**Electronic supplementary material:**

The online version of this article (10.1186/s13049-019-0642-2) contains supplementary material, which is available to authorized users.

## Introduction

Sepsis is a common and potentially life threatening response to an infection [1]. Worldwide there are an estimated 31.5 million cases of uncomplicated sepsis and 19.4 million cases of severe sepsis or septic shock resulting in 5.3 million deaths each year [2]. The majority of these cases originate in the community and will present to hospital via the Emergency Department (ED) [3, 4]. More than half of ED sepsis cases will arrive via Emergency Medical Services (EMS) [5–10]. These patients are likely to be sicker than those arriving by other means [6, 8–11].

International guidelines for sepsis advocate that treatment be initiated at the earliest possible opportunity [1, 12]. Recent data suggest each hour delay to antibiotic therapy results in an increase in mortality among patients with septic shock of 2.8% [13] whereas for each hour delay in delivering a 3 h resuscitation bundle (intravenous antibiotics, vascular therapy and obtaining blood cultures) sees a 4% increase in mortality [14]. Early EMS intervention has helped to improve outcomes for other time critical, life-threatening conditions such as acute myocardial infarction [15], stroke [16] and major trauma [17]. It remains to be seen if early EMS intervention in sepsis improves outcomes.

Small observational studies indicate prehospital care reduces time to antibiotics for patients with sepsis, without improving clinical outcomes [5, 18, 19]. Thus far, trials of prehospital antibiotics have failed to demonstrate improved clinical outcomes [20, 21]. One potential reason for this is inclusion of low acuity sepsis patients within prehospital studies [21].

Despite frequent exposure to patients with potentially life-threatening sepsis [22], prehospital recognition of sepsis is challenging [18, 23–27]. Indeed, a recent analysis of 240 patients transported by Ambulance Victoria, who were subsequently enrolled in the ARISE study, showed that despite the presence of demonstrable physiologic abnormalities, only 165 patients had documentation of infection in their prehospital record [28]. There are several reasons why this may be so, including, suboptimal teaching and understanding of the condition [6, 29–31], encountering sepsis cases earlier in the disease process when the clinical presentation is less obvious [32], lack of in-hospital diagnostic capability [18] and dependence upon SIRS criteria to formulate a diagnosis [33, 34]. Reliance upon paramedic gestalt may therefore mean patients with significant pathology are not identified until after arrival at hospital. It has been argued that a prehospital sepsis screening tool to assist prehospital clinicians identify ‘sick’ sepsis patients would be helpful [6, 35].

The NICE guideline “Recognition and management of sepsis (NG51)”, stratifies the risk of “severe illness or death from sepsis” (see Table 1) [12]. It recommends that patients categorised as “high risk of severe illness or death from sepsis” should receive antibiotics within 1 h [12]. The aim of this study was to develop a simple scoring system that would help identify those adult patients who might benefit most from early intervention. For brevity we refer to the NICE categorisation of “high risk of severe illness or death from sepsis” as ‘high risk’.

**Table 1:** NICE Risk of severe illness or death from sepsis

Category	High risk criteria	Moderate to high risk criteria	Low risk criteria
History	Objective evidence of new altered mental state	History from patient, friend or relative of new onset of altered behaviour or mental state History of acute deterioration of functional ability Impaired immune system (illness or drugs including oral steroids) Trauma, surgery or invasive procedures in the last 6 weeks	Normal behaviour
Respiratory	Raised respiratory rate: 25 breaths per minute or more New need for oxygen (more than 40% FiO2) to maintain saturation more than 92% (or more than 88% in known chronic obstructive pulmonary disease)	Raised respiratory rate: 21–24 breaths per minute	No high risk or moderate to high risk criteria met
Blood pressure	Systolic blood pressure 90 mmHg or less or systolic blood pressure more than 40 mmHg below normal	Systolic blood pressure 91–100 mmHg	No high risk or moderate to high risk criteria met
Circulation and hydration	Raised heart rate: more than 130 beats per minute Not passed urine in previous 18 h. For catheterised patients, passed less than 0.5 ml/kg of urine per hour	Raised heart rate: 91–130 beats per minute (for pregnant women 100–130 beats per minute) or new onset arrhythmia Not passed urine in the past 12–18 h For catheterised patients, passed 0.5–1 ml/kg of urine per hour	No high risk or moderate to high risk criteria met
Temperature		Tympanic temperature less than 36 °C	
Skin	Mottled or ashen appearance Cyanosis of skin, lips or tongue Non-blanching rash of skin	Signs of potential infection, including redness, swelling or discharge at surgical site or breakdown of wound	No non-blanching rash

## Methods

### Study design and population

This study was conducted and reported consistent with TRIPOD reporting guidelines [36]. We utilised a retrospective sample of consecutive adult patients (age ≥ 18 years) transported by West Midlands Ambulance Service NHS Foundation Trust (WMAS) to Royal Stoke University Hospital NHS Trust (previously University Hospital North Staffordshire NHS Foundation Trust) between 01 July 2013 and 30 June 2014. Exclusion criteria were age under 18 years, cardiac arrest, trauma or mental health aetiology (determined from hospital discharge diagnosis). No interventions were undertaken as part of this study.

### Patient involvement

A study committee was convened to oversee the Clinical Doctoral Research Fellowship awarded to MAS. This committee included a patient representative who contributed to the initial research plan, and commented on chapters of the doctoral thesis. The patient representative did not contribute to writing or reviewing this manuscript.

### Primary outcome measure

The primary outcome measure was categorisation as ‘high risk of severe illness or death from sepsis’, as per the National Institute for Health and Care Excellence (NICE) Guideline “Recognition and management of sepsis (NG51)” [12], on arrival at the ED. For each included patient, category of ‘risk of severe illness or death from sepsis’ was assigned as ‘no risk’ i.e. no infection present, ‘low risk’, ‘moderate risk’ or ‘high risk’, dependent upon presence of infection and presenting vital signs. Presence of infection was determined using the ED discharge diagnosis. Classification of the risk of severe illness or death from sepsis was determined utilising clinical data recorded in the ED in accordance with Table 1.

### Record linkage

LinkPlus® software (version 3.0 beta, Centres for Disease Control and Prevention Cancer Division, Atlanta, Georgia), a probabilistic linkage program was used to link ambulance and ED records. First name, surname, gender, date of birth, home address post code and incident date were used to link records. All candidate record pairs were manually reviewed. Following linkage patient identifiable data were deleted.

### Missing data

Statistical analyses were performed using R (version 3.3.1) in R Studio (version 0.99.903). Missing data were processed by multiple imputation using the R package Multiple Imputation by Chained Equations (MICE) (version 2.25) [37] with a Fully Conditional Specification. To ensure robust imputation of missing values, the number of imputed datasets required was slightly higher than the percentage of cases with incomplete data. For example if 18% of cases had incomplete data 20 imputations would be required. Variables that were functions of another variable were not imputed, rather their component variables were imputed and the function determined after imputation. For example, Glasgow Coma Score (GCS) sum is a function of three variables GCS eye, GCS verbal and GCS motor. GCS sum was not imputed, rather GCS eye, GCS verbal and GCS motor were imputed and GCS sum was calculated from the component values.

Satisfactory imputation of missing data was confirmed by inspection of both convergence plots and density plots of imputed values and observed data (R package MICE (version 2.25) [37] as well as calculation of R-hat convergence statistics using the R package MICEadds (version 1.9–0) [38].

### Model development

Following the imputation of missing data we developed a multivariable logistic model for high risk of severe illness or death from sepsis (‘high risk’) on arrival at the ED using several steps. First, the data were divided into derivation and validation cohorts using the R package Caret (version 6.0–71) [39]. The number of cases assigned to the derivation and validation cohorts were based upon recommendations by Harrell, Royston, Steyerberg and Vergouwe [40–47]. It has been argued that, when developing a predictive model, at least ten instances of the outcome of interest are required, per candidate predictor included in the model, to ensure statistically valid results [40–46]. Similarly, Vergouwe et al [47] argue that at least 100 events and 100 non-events are required to assess model performance in the validation dataset. However, Steyerberg [45] suggests that, to detect small differences in model performance, the validation dataset should contain at least 250 cases of the outcome of interest.

Derivation of the SEPSIS score was undertaken using the derivation dataset. We assessed the quality of candidate predictor variables using univariable logistic regression. Then, we constructed candidate parsimonious multivariable logistic models. Next, we assigned weighted point scores to included predictor variables. Thereafter we compared the performance of candidate models. Finally, we undertook internal validation of the SEPSIS score using the validation dataset.

Simple logistic regression was undertaken in an attempt to quantify the relationship between individual candidate predictor variables and the primary outcome measure (‘high risk’). It is common at this stage to exclude variables that are not statistically associated with the outcome of interest however we did not, as to do so may exclude clinically important variables [45]. Candidate predictor variables were assessed for multicollinearity using the R package Caret (version 6.0–71) [39], any variables with a correlation coefficient above 0.9 (positive or negative) were considered to be highly collinear. Inclusion of multiple variables with high collinearity was avoided, either by exclusion of redundant variables, or by generation of parallel candidate models that did not contain multiple highly collinear variables.

Selection of independent predictor variables was informed by previously demonstrated clinical usefulness and by backward stepwise selection using the Akaike Information Criterion (AIC) and the Wald test *p*-value. Relative performance of candidate models was assessed by determining the AIC and Brier Score for each model.

Many paramedics will not have access to resources to calculate a complex model in clinical practice. Therefore, to simplify the models for use at the roadside, continuous variables were transformed into categorical variables by subdividing the variable range into intervals.

Variable intervals were determined by visual inspection of Loess curves and cut points were calculated using R the package OptimalCutPoints (version 1.1–3) [48]. We also considered normal physiologic ranges and intervals utilised in alternate sepsis screening tools. We recognise that conversion of continuous variables to categorical variables results in loss of precision. To guard against the loss of precision, continuous predictor variables were initially subdivided into multiple small intervals. Weighted scores were assigned to each interval by rounding the regression coefficient for each interval to the nearest integer. Intervals with equally weighted scores were subsequently merged to generate fewer, wider intervals, to simplify use by bed-side clinicians.

### Model performance

Model performance was assessed using the validation dataset. Model calibration (goodness of fit) was assessed by calculation of the calibration slope (R package ResourceSelection (version 0.3–1)) [49]. The calibration slope is a graphical assessment of the relationship between predicted and observed outcomes [50], with predictions represented on the x-axis, and outcomes represented on the y-axis. Perfect predictions fall on the 45^°^ line (calibration slope = 1) [51]. The Hosmer-Lemeshow goodness of fit test was not used to assess goodness of fit as a significant result, suggesting inadequate fit, is common when using large datasets [52]. Model discrimination was assessed by calculating the area under the receiver operating characteristic curve (AUROC) (R package ROCR (version 1.0–5) [53]. Model performance was assessed by calculating sensitivity, specificity, positive predictive value, negative predictive value, positive likelihood ratio and negative likelihood ratio (R package epiR (version 0.9–77) [54]).

### Ethical approval

Permission to access patient identifiable data without consent was granted by the Health Research Authority (HRA) Confidentiality Advisory Committee (CAG) (CAG 4–03(PR2)2014). A favourable ethical opinion was obtained from the National Research Ethics Service (NRES) Committee South Central - Oxford C (14/SC/0163). Data storage and handling were conducted in accordance with WMAS standard operating procedures.

## Results

**Fig. 1:**
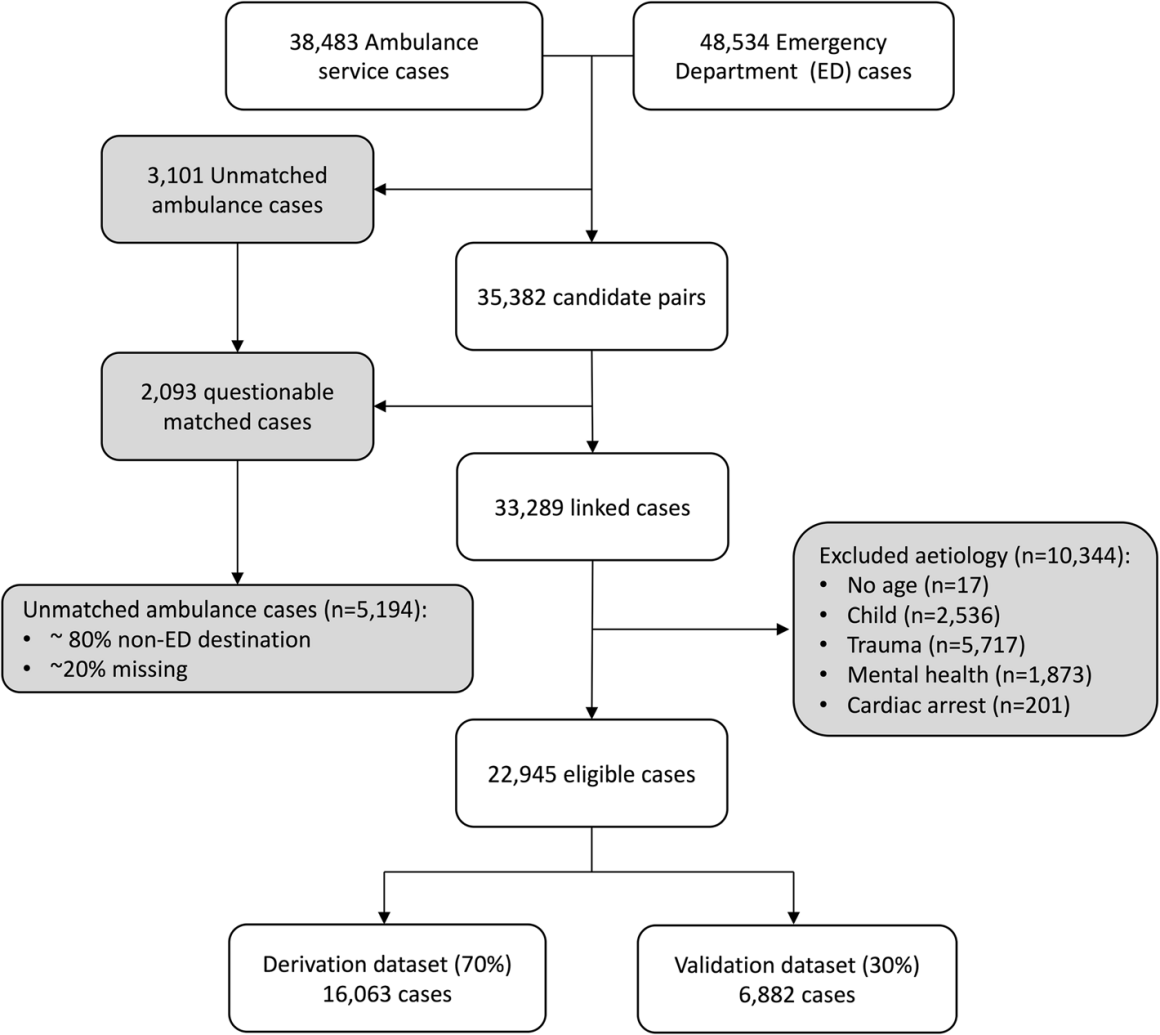
Included cases

From 38,483 unique ambulance records, LinkPlus® generated 35,382 candidate record pairs. Manual review of all candidate pairs confirmed 33,289 (86.5%) were correctly linked with their corresponding ED record. Following removal of excluded case aetiologies, 22,945 cases remained (see Fig. 1). Of the 5,194 (13.5%) unlinked ambulance cases, a significant proportion were transported to hospital destinations other than the ED, for example the Medical Admission Unit (MAU). An initial review of 120 unlinked cases confirmed that 97 (80.8%) were transported to hospital destinations other than the ED. A small proportion of unlinked cases result from an ambulance crew being unable to identify a patient in the early stages of their health care episode, for example when a patient is unconscious and their name cannot be determined. There were 58 instances (1.1% of unlinked cases) where the name or surname fields of the ambulance record were “missing” or “unknown”.

Of 54 potential variables, 30 were deemed inappropriate for inclusion in the model (Additional file 1: Table S1). Of 24 included variables, four (blood sugar, temperature, capillary bed refill time (CBRT) and skin) had greater than 10% missing values; and four (left pupil reaction, left pupil size, right pupil reaction and right pupil size) had between 5 and 10% missing values. The remaining 16 variables had fewer than 2% missing values. There were no cases where the ED discharge diagnosis field was empty.

Of the 22,945 included cases, only 12,517 (54.6%) had complete data, all other cases had at least one missing data point. To ensure robust imputation 50 imputed datasets were generated. Convergence plots (Additional file 1: Figure S1), density plots (Additional file 1: Figure S2), Box and whisker plots (Additional file 1: Figure S3) and R-hat statistics (Additional file 1: Table S2) indicate that healthy convergence was achieved for all imputed variables except Left Pupil Size and Right Pupil size.

The dataset used comprised 24 variables, therefore to calculate reliable estimates, the derivation dataset must include at least 240 instances of ‘high risk’. The imputed dataset was divided into a derivation dataset of 16063 cases (70%) and a validation dataset of 6882 cases (30%). The derivation dataset contained 593 instances of ‘high risk’ (3.7%), sufficient cases to accommodate a model with 59 variables. The validation dataset contained 254 instances of ‘high risk’ (3.7%), sufficient cases to accommodate 25 variables and to detect small differences in model performance [45]. Patient characteristics were consistent across derivation and validation cohorts (Table 2).

**Table 2:** Patient characteristics

Variable	Derivation *n* = 16,063 (70%)	Validation *n* = 6,882 (30%)
Location		
Home, n (%)	11,408 (71)	4,964 (72)
Nursing home, n (%)	1,028 (6)	414 (6)
Other, n (%)	3,627 (23)	1,504 (22)
Age (years), mean (SD)	63 (21)	62 (21)
Gender		
Male, n (%)	7,884 (49)	3,346 (49)
Respirations (breaths/min), mean (SD)	20 (6)	20 (6)
Oxygen saturation (%), mean (SD)	96 (5)	96 (5)
Heart rate (beats/min), mean (SD)	92 (24)	92 (24)
Systolic blood pressure (mmHg), mean (SD)	133 (27)	133 (27)
Diastolic blood pressure (mmHg), mean (SD)	78 (17)	78 (17)
Temperature (°C), mean (SD)	36.8 (0.9)	36.8 (0.9)
Blood sugar (mmol/L), mean (SD)	7.0 (3.4)	7.0 (3.3)
Glasgow Coma Score, median (IQR)	15 (15–15)	15 (15–15)
Capillary bed refill time		
Normal (< 2 s), n (%)	15,319 (95)	6,567 (95)
Delayed (> 2 s), n (%)	744 (5)	315 (5)
Skin		
Normal, n (%)	10,366 (73)	5,320 (77)
Pallor, n (%)	2,669 (19)	1,037 (15)
Flushed, n (%)	856 (6)	359 (5)
Cyanosed, n (%)	181 (1)	83 (1)
Jaundice, n (%)	92 (0.6)	51 (0.7)
Mottled, n (%)	53 (0.4)	20 (0.2)
Rash, n (%)	21 (0.1)	12 (0.1)
Pupil size (mm), median (IQR)	3 (3–4)	3 (3–4)
Pupil reaction		
Brisk, n (%)	13,447 (93)	6,462 (94)
Sluggish, n (%)	923 (6)	381 (6)
Fixed, n (%)	128 (0.8)	39 (0.5)
NICE risk		
No risk (no infection), n (%)	13,083 (81.4)	5,607 (81.5)
Infection, n(%)	2980 (18.6)	1275 (18.5)
Low risk, n (%)	1,048 (6.5)	448 (6.5)
Moderate risk, n (%)	1,339 (8.3)	573 (8.3)
High risk, n (%)	593 (3.7)	254 (3.7)

Univariable logistic regression analysis identified the following variables to be statistically significant predictors of ‘high risk’ in the derivation dataset: EMS impression, location, age, respirations, oxygen saturations (SpO2), pulse, systolic blood pressure (SBP), diastolic blood pressure (DBP), temperature, blood sugar (BM), Skin, CBRT, left pupil reaction, right pupil reaction, left pupils size, right pupil size, GCS sum, GCS eye, GCS verbal, GCS motor, AVPU score (Additional file 1: Tables S3 & S4).

A perfect correlation was identified between left and right pupil reactions, and a near perfect correlation was noted between left and right pupil size (Additional file 1: Figure S4). Differences in pupil size or pupil reactions are not known to be associated with sepsis. To avoid issues arising from inclusion of highly correlated variables, data concerning the left pupil were excluded from further analysis.

Strong correlations between GCS sum, GCS components (GCS eye, GCS verbal and GCS motor) and AVPU score (used to document level of consciousness) were identified. It is unclear which measure of level of consciousness would generate the most effective predictive model of sepsis. Three candidate models, using GCS sum, GCS components and AVPU score as their respective measure of consciousness, were generated.

Multivariable logistic regression analysis identified the following variables to be significant: location, age, respirations, oxygen saturations, pulse rate, systolic blood pressure, temperature, skin colour and level of consciousness. The number of instances each variable was selected, and the related Wald test statistic, for each model is reported in Table 3.

**Table 3:** Selection of variables for inclusion in multivariable models

Predictor variable	Incidence of variable selection			Wald test *p*-value		
	*GCS (sum) model*	*GCS (components) model*	*AVPU model*	*GCS (sum) model*	*GCS (components) model*	*AVPU model*
Location	50	50	50	0.002	0.004	0.003
Age	50	50	50	< 0.001	< 0.001	< 0.001
Gender	0	0	0	–	–	–
Resps	50	50	50	< 0.001	< 0.001	< 0.001
SpO2	50	50	50	< 0.001	< 0.001	< 0.001
Pulse	50	50	50	< 0.001	< 0.001	< 0.001
SBP	50	50	50	< 0.001	< 0.001	< 0.001
DBP	0	0	0	–	–	–
Temp	50	50	50	< 0.001	< 0.001	< 0.01
BM	1	1	0	0.86^*^	0.85^*^	0.87^*^
Skin	50	50	50	0.007	0.007	0.014
CBRT	0	0	0	–	–	–
RPupilReact	11	12	8	0.42^*^	0.41^*^	0.46^*^
RPupilSize	0	0	0	–	–	–
GCS_sum	50	NA	NA	< 0.001	NA	NA
GCS_eye	NA	0	NA	NA	–	NA
GCS_verbal	NA	50	NA	NA	< 0.001	NA
GCS_motor	NA	0	NA	NA	–	NA
AVPU	NA	NA	50	NA	NA	< 0.001

*SpO2* peripheral oxygen saturation, *SBP* systolic blood pressure, *DBP* diastolic blood pressure, *Temp* temperature (C), *BM* blood sugar mmol/L, *CBRT* capillary bed refill time, *GCS* Glasgow coma score, *AVPU* alert, verbal, pain or unresponsive

^*^Did not reach statistical significance

Categorisation of continuous variables is summarised in Table 4. Simple weighted scores to enable calculation of the SEPSIS score were obtained by rounding regression coefficients to the nearest integer (Additional file 1: Tables S5, S6 & S7). Intervals for continuous variables with the same weighted score were merged to simplify calculation of the SEPSIS score (Additional file 1: Tables S8, S9 & S10). The SEPSIS score is defined as the sum of the simplified weighted scores for each variable.

**Table 4:** Continuous variable intervals

Variable	Interval
Age	below 40 (reference)
	40 to 49
	50 to 59
	60 to 69
	70 to 79
	80 to 89
	90 to 99
	100 plus
Respirations	below 10
	10 to 20 (reference)
	21 to 25
	26 to 30
	31 to 35
	36 to 40
	41 to 45
	46 to 50
	51 to 55
	56 to 60
	60 plus
Pulse	below 60
	60 to 100 (reference)
	101 to 110
	111 to 120
	21 to 130
	131 to 140
	141 to 150
	151 to 160
	161 to 170
	171 to 180
	180 plus
SBP	below 60
	60 to 69
	70 to 79
	80 to 89
	90 to 99
	100 to 120 (reference)
	121 to 129
	130 to 139
	140 to 149
	150 to 159
	160 plus
SpO2	above 93 (reference)
	89 to 93
	85 to 88
	below 85
Temperature	below 35
	35.0 to 35.5
	35.6 to 36.0
	36.1 to 36.5
	36.6 to 37.4 (reference)
	37.5 to 38.0
	38.1 to 38.5
	38.6 to 39.0
	39.1 to 39.5
	39.6 to 40.0
	above 40
GCS	3–9
	10–12
	13–14
	15 (reference)

*SBP* systolic blood pressure, *SpO2* peripheral oxygen saturations, *GCS* Glasgow coma score

Relative performance of the three parallel models is reported in Table 5. The model utilising GCS sum as the measure of consciousness was calculated to have the best performance statistically. The final parsimonious model, with merged intervals and weighted scores, is reported in Table 6.

**Table 5:** Comparison of models

Model	AIC statistic	Brier score
Model using GCS sum	2,854.1	0.0321
Model using GCS components	2,864.4	0.0321
Model using AVPU	3,315.0	0.0325

*GCS* Glasgow coma score, *AVPU* alert, verbal, pain or unresponsive

**Table 6:** SEPSIS score

		95% CI			Score assigned
Variable	ß_i_	lower	upper	*p* value	
Age					
Below 40	reference				0
40 to 60	0.35	−0.15	0.86	0.17	0
Over 60	0.94	0.51	1.36	< 0.001	1
Respirations					
Below 10	−12.44	− 559.61	534.73	0.96	0
10 to 20	reference				0
21 to 40	0.90	0.66	1.14	< 0.001	1
40 to 60	1.72	1.26	2.18	< 0.001	2
60 plus	−11.77	− 1970.04	1946.51	0.99	0
SpO2					
Above 93	reference				0
Below 94	1.03	0.80	1.26	< 0.001	1
Pulse					
Below 60	−0.56	−1.40	0.28	0.19	0
60 to 100	reference				0
101 to 140	0.75	0.51	0.99	< 0.001	1
141 to 160	1.67	1.27	2.08	< 0.001	2
Over 160	0.60	−0.16	1.35	0.12	0
SBP					
Below 60	0.50	−1.53	2.52	0.63	0
60 to 99	0.65	0.33	0.97	< 0.001	1
100 to 120	reference				0
121 to 160	−0.21	−0.47	0.05	0.11	0
Over 160	−0.72	−1.10	−0.34	< 0.001	−1
GCS (sum)					
15	reference				0
13 to 15	−0.13	−0.48	0.21	0.45	0
3 to 12	0.78	0.47	1.09	< 0.001	1
Temperature					
Below 36.6	−0.20	−0.48	0.09	0.18	0
36.6 to 37.4	reference				0
37.5 to 39.5	0.97	0.71	1.23	< 0.001	1
Above 39.5	1.71	1.23	2.18	< 0.001	2
Skin					
Normal	reference				0
Jaundice, pallor, mottling	0.51	0.27	0.75	< 0.001	1
Any other	0.23	−0.08	0.55	0.14	0
			Maximum score		11

*SBP* systolic blood pressure, *SpO2* peripheral oxygen saturations, *GCS* Glasgow coma score

**Fig. 2:**
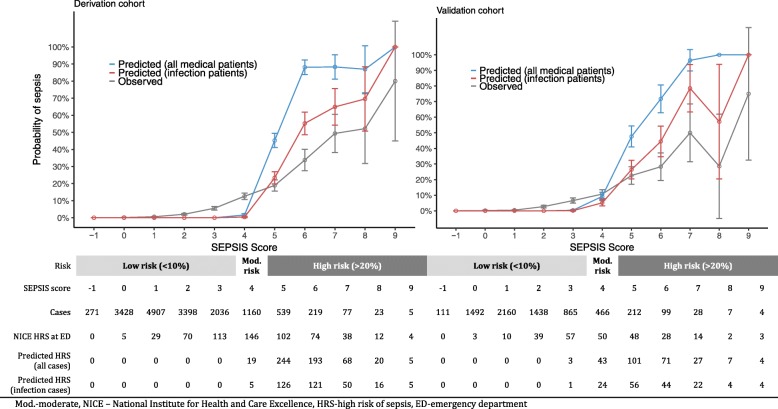
Observed vs expected probability of sepsis. Mod.-moderate, HRS-high risk of severe illness or death from sepsis, ED-emergency department

The calibration slope for the derivation and validation datasets was 1.0 and 0.97 respectively, suggesting the SEPSIS score has adequate fit. The AUROC was 0.87 (95% CI 0.85–0.88) for the derivation dataset and 0.86 (95% CI 0.84–0.88) for the validation dataset. We report performance measures for each point score of the SEPSIS score in Additional file 1: Table S11, and categorise patients as low likelihood (< 10%), moderate likelihood (10–20%) or high likelihood (> 20%) by applying different thresholds for the SEPSIS score (see Fig. 2).

Where the SEPSIS score indicates greater than 20% likelihood of ‘high risk’ at ED (SEPSIS score ≥ 5), we observed satisfactory performance characteristics as reported in Table 7. If, as per the NICE sepsis guideline [NG51], the presence of infection is required before a diagnosis of sepsis can be made [12], then in the validation cohort of 6882 adult patients, 195 (2.8%) patients were classified as being having ‘high risk’, and 6687 (97.2%) were classified as not having ‘high risk’. Among those patients classified as ‘high risk’, 95 (48.7%) did have ‘high risk’ (true positive), while 100 (51.3%) patients had their risk of sepsis overestimated i.e. were incorrectly identified as having ‘high risk’ (false positive). Within the 100 false positive cases, 79 (40.5%) patients had ‘moderate risk’ and 21 (10.8%) had ‘low risk’ on arrival at the ED. Among patients identified as not septic, 6528 (95.6%) did not have sepsis (true negative), while 159 (62.6%) did have ‘high risk’ on arrival at the ED but were incorrectly classified by the SEPSIS score as not having sepsis (false negatives).

**Table 7:** Comparison of performance between datasets (SEPSIS score ≥ 5)

Estimate	Derivation cohort *n* = 16063	Validation cohort *n* = 6882
Undifferentiated medical cases (estimate(95% confidence interval))		
Sensitivity	0.39 (0.35–0.43)	0.37 (0.31–0.44)
Specificity	0.96 (0.96–0.96)	0.96 (0.96–0.97)
Positive predictive value	0.27 (0.24–0.30)	0.27 (0.23–0.32)
Negative predictive value	0.98 (0.97–0.98)	0.98 (0.97–0.98)
Positive likelihood ratio	9.48 (8.35–10.76)	9.72 (7.96–11.87)
Negative likelihood ratio	0.64 (0.60–0.68)	0.65 (0.59–0.72)
Infection present (estimate(95% confidence interval))		
Sensitivity	0.39 (0.35–0.43)	0.37 (0.31–0.44)
Specificity	0.99 (0.98–0.99)	0.98 (0.98–0.99)
Positive predictive value	0.50 (0.45–0.55)	0.49 (0.42–0.56)
Negative predictive value	0.98 (0.97–0.98)	0.98 (0.97–0.98)
Positive likelihood ratio	26.1 (22.2–30.1)	24.8 (19.3–31.9)
Negative likelihood ratio	0.62 (0.58–0.66)	0.64 (0.58–0.70)

## Discussion

Screening tool performance is commonly described in terms of sensitivity, specificity, positive predictive value (PPV) and positive likelihood ratio (PLR). Sensitivity describes the ability of a test to correctly identify those with the disease (true positive rate), whereas specificity describes the ability of a test to correctly identify those without the disease (true negative rate). PPV represents the proportion of patients with positive test who actually have the disease, while the PLR shows how much more likely someone is to get a positive test if he/she has the disease, compared with a person without disease [55].

The performance characteristics of several existing screening tools used to support paramedic recognition of sepsis are reported in Table 8.

**Table 8:** Reported performance of alternate screening tools

Screening tool	Sensitivity (95% CI)	Specificity (95% CI)	PPV (95% CI)	NPV (95% CI)
PreSS				
Polito [56]	0.85 (NR)	0.47 (NR)	0.19 (NR)	0.96 (NR)
PreSep				
Bayer [57]	0.85 (0.77–0.92)	0.86 (0.82–0.90)	0.63 (NR)	0.95 (NR)
Jouffrey [58]	0.92 (NR)	0.29 (NR)	0.41 (NR)	0.88 (NR)
Robson tool (severe sepsis)				
McClelland [24]	0.30 (0.12–0.47)	0.77 (0.60–0.95)	NR	NR
Wallgren [27]	0.93 (NR)	NR	NR	NR
Robson tool (sepsis)				
McClelland [24]	0.43 (0.28–0.58)	0.14 (0.0–0.40)	NR	NR
Bayer [57]	0.95 (NR)	0.43 (NR)	0.32 (NR)	0.97 (NR)
Wallgren [27]	0.75 (NR)	NR	NR	NR
Dorsett [59]	0.47 (0.31–0.62)	0.80 (0.71–0.87)	NR	NR
qSOFA				
Dorsett [59]	0.16 (0.07–0.31)	0.97 (0.92–0.99)	NR	NR
Jouffroy [58]	0.62 (NR)	0.16 (NR)	0.29 (NR)	0.44 (NR)
Tusgul (ICU admission) [60]	0.36 (0.27–0.47)	NR	NR	NR

*CI* confidence interval, *NR* not reported, *ICU* intensive care unit

Existing data may suggest the PreSep score is the best performing sepsis screening tool. However, when applied to the same validation dataset used to test the SEPSIS score, the PreSep score remains more sensitive (0.61 (95%CI 0.55–0.67)), but has poorer specificity 0.95 (95%CI 0.95–0.96), PPV 0.33 (95%CI 0.29–0.37) and PLR 12.76 (95%CI 11.03–14.76). These data suggest the PreSep score may not in fact be the most useful for identifying those patients at risk of severe illness or death from sepsis by the bedside paramedic.

In this work, a threshold of SEPSIS score ≥ 3 has a sensitivity of 0.80 (95CI 0.74–0.84) specificity of 0.93 (95%CI 0.93–0.94), PPV of 0.32 (95%CI 0.28–0.36) and PLR of 12.17 (95%CI 10.90–13.59). Adopting a threshold of SEPSIS score ≥ 5 has a sensitivity of 0.37 (95%CI 0.31–0.44) specificity of 0.98 (95%CI 0.98–0.99), PPV of 0.49 (95%CI 0.42–0.56) and PLR of 24.8 (95%CI 19.3–31.9). In terms of patients, a SEPSIS score ≥ 3 correctly identified 202 patients with ‘high risk’, missed 52 patients and incorrectly identified that 433 had sepsis when in fact they did not. When adopting a threshold of SEPSIS score ≥ 5 95 patients with ‘high risk’ were correctly identified and 159 patients missed and the number of patients incorrectly classified as ‘high risk’ was lower at 100.

Deciding what threshold to adopt for a ‘positive’ identification is a system level decision. Many systems will favour sensitivity to ensure cases are not missed but this needs to be balanced against significant over triage and the impact on resources this may have. Indeed, previous definitions for sepsis have been criticised for being overly sensitive with inadequate specificity [33, 34]. On this basis we suggest adopting a cut-off SEPSIS≥5 to favour specificity and reduce false positive cases as reflected in the increased PLR. However, we recognise that many systems/clinicians may prefer to adopt a lower cut-off to increase sensitivity.

Potentially important variables have been omitted from the SEPSIS score. Lactate is commonly used to help stratify severity among patients with sepsis [12, 61]. Lactate is not measured by ambulance crews in the participating ambulance service, therefore it was not available for consideration during SEPSIS score development. However, it has been reported that inclusion of prehospital lactate does not improve prehospital identification of sepsis [62]. Secondly, Hunter et al. argued that end-tidal carbon dioxide (EtCO_2_) measured by EMS was an important predictor of sepsis, severe sepsis and mortality, reporting an AUROC of 0.99 (95%CI 0.99–1.0), 0.80 (95%CI 0.73–0.86) and 0.70 (95%CI 0.57–0.83) respectively [63]. Although EtCO_2_ can be measured by EMS personnel in the participating ambulance service, it is currently only measured when undertaking advanced airway interventions. It was thus seldom available for consideration in the SEPSIS score. It remains to be seen if inclusion of either of these variables would improve the performance of the SEPSIS score.

Stratification of the likelihood of ‘high risk’ on arrival at the ED, using the SEPSIS score, may help inform the provision of prehospital care, and/or the destination to which the patient is transferred. Although a SEPSIS score ≥ 5 has high specificity, careful consideration is warranted before utilising the SEPSIS score to initiate treatment. Current evidence does not support routine prehospital administration of antibiotics [21]. In addition, appropriate antibiotic stewardship, and the need to obtain venous blood samples to culture pathogens prior to antibiotic administration, must be considered before implementing intervention strategies.

Ours is the first study to develop a prehospital sepsis screening tool using data from a UK ambulance service and the first to utilise the NICE high risk of severe illness or death from sepsis classification as the primary outcome measure. Ours is also the only such study to employ multiple imputation to manage missing data. A strength of this study is the primary outcome measure was determined using objective data from the ED record, rather than ED clinician diagnosis or International Classification of Disease (ICD) code, maximising specificity for the outcome measure. However, the SEPSIS score has been derived and internally validated with a retrospective data sample from a single centre which limits generalisability of the findings. In addition, it has not yet been clinically demonstrated that patients with high risk of serious illness or death from sepsis, as per the NICE guideline, benefit from early antibiotic therapy.

## Conclusion

We derived and internally validated a prehospital model that predicts risk of severe illness or death from sepsis as per NICE guideline NG51 on arrival at the ED. We used routine EMS data, linked to ED records, in a heterogeneous medical population, to develop the SEPSIS score. The SEPSIS score could be a valuable tool for identifying sepsis patients in need of early antibiotic therapy. It requires external validation and assessment of performance when in use by ambulance clinicians.

## Additional file

Additional file 1: **Table S1.** Excluded candidate predictor variables. **Table S2** R-Hat statistics. **Table S3.** Logistic Regression of continuous candidate predictor variables. **Table S4.** Logistic regression of categorical candidate predictor variables. **Table S5.** Multivariable logistic regression model utilising GCS sum **Table S6.** Multivariable logistic regression model utilising GCS components. **Table S7.** Multivariable logistic regression model utilising AVPU. **Table S8.** Final weighted scores for candidate model using GCS sum. **Table S9.** Final weighted scores for candidate model using GCS components. **Table S10.** Final weighted scores for candidate model using AVPU. **Table S11.** Operating characteristics for the SEPSIS score. **Figure S1.** Convergence plots. **Figure S2.** Density plots. **Figure S3.** Box and whisker plots. **Figure S4.** Colinearity between candidate predictor variables. (DOCX 3170 kb)
