# Supplementary material for: Derivation and internal validation of the screening to enhance prehospital identification of sepsis (SEPSIS) score in adults on arrival at the emergency department
Source: Scand J Trauma Resusc Emerg Med. 2019 Jul 16;27:67. doi: 10.1186/s13049-019-0642-2 (PMC6636043; doi:10.1186/s13049-019-0642-2)
Supplement: Supplementary file 1 — Table S1. Excluded candidate predictor variables. Table S2 R-Hat statistics. Table S3. Logistic Regression of continuous candidate predictor variables. Table S4. Logistic regression of categorical candidate predictor variables. Table S5. Multivariable logistic regression model utilising GCS sum Table S6. Multivariable logistic regression model utilising GCS components. Table S7. Multivariable logistic regression model utilising AVPU. Table S8. Final weighted scores for candidate model using GCS sum. Table S9. Final weighted scores for candidate model using GCS components. Table S10. Final weighted scores for candidate model using AVPU. Table S11. Operating characteristics for the SEPSIS score. Figure S1. Convergence plots. Figure S2. Density plots. Figure S3. Box and whisker plots. Figure S4. Colinearity between candidate predictor variables. (DOCX 3170 kb) [file 13049_2019_642_MOESM1_ESM.docx]

**Additional file 1**

| **Table S1** Excluded candidate predictor variables | | | | | | |  |
| --- | --- | --- | --- | --- | --- | --- | --- |
| **Variable** | | **Reason for exclusion** | | | | | |
| PatientID | | Unique ID. Not patient/clinically related | | | | | |
| UHNS_ID | | Unique ID. Not patient/clinically related | | | | | |
| Dispatch Complaint1 | | Not patient/clinically related | | | | | |
| Dispatch Complaint2 | | Not patient/clinically related | | | | | |
| Dispatch Category | | Not patient/clinically related | | | | | |
| Forename | | PID removed following linkage | | | | | |
| Surname | | PID removed following linkage | | | | | |
| Address 1 | | PID removed following linkage | | | | | |
| Address 2 | | PID removed following linkage | | | | | |
| City | | PID removed following linkage | | | | | |
| County | | PID removed following linkage | | | | | |
| Post Code | | PID removed following linkage | | | | | |
| DOB | | Age variable calculated in preference | | | | | |
| Incident Address | | PID removed following linkage | | | | | |
| Incident City | | PID removed following linkage | | | | | |
| Incident Postcode | | PID removed following linkage | | | | | |
| Incident Time | | Not patient/clinically related | | | | | |
| Response Priority | | Not patient/clinically related | | | | | |
| Mobile | | Not patient/clinically related | | | | | |
| Arrive Scene | | Not patient/clinically related | | | | | |
| Leaving Scene | | Not patient/clinically related | | | | | |
| Destination Arrival | | Not patient/clinically related | | | | | |
| Peak Flow (l/min) | | Very high missingness | | | | | |
| End Tidal CO2 (kPa) | | Very high missingness | | | | | |
| Airway Signs | | Very high missingness | | | | | |
| Airway Status | | Very high missingness | | | | | |
| Arrest Occurance | | Excluded case type | | | | | |
| Crew Level 1 | | Not patient/clinically related | | | | | |
| Crew Level 2 | | Not patient/clinically related | | | | | |
| Institution Name | | Not patient/clinically related | | | | | |
| PID – personal identifiable data | | | | | | | |
| \| **Table S2** R-Hat statistics \| \| \| \| \| --- \| --- \| --- \| --- \| \| **Variable** \| **% Missing** \| **Mean** \| **Variance** \| \| Imp \| 0.9762475 \| 1.0016182 \| 1.0047219 \| \| Resps \| 0.2571366 \| 0.9991814 \| 1.0523770 \| \| SpO2 \| 0.7103944 \| 1.0026238 \| 1.0008604 \| \| Pulse \| 0.5665722 \| 1.0037685 \| 0.9977388 \| \| SBP \| 1.7127915 \| 1.0248377 \| 1.0073032 \| \| DBP \| 1.8609719 \| 1.0253729 \| 1.0076894 \| \| Temp \| 15.4281979 \| 1.0011967 \| 0.9988709 \| \| BM \| 22.8285029 \| 1.0017126 \| 1.0002574 \| \| GCS_eye \| 1.3161909 \| 1.0092198 \| 1.0081991 \| \| GCS_verbal \| 1.3161909 \| 1.0126162 \| 1.0070718 \| \| GCS_motor \| 1.3161909 \| 1.0095771 \| 1.0100301 \| \| Skin \| 11.4970582 \| 0.9984954 \| 0.9984608 \| \| CBRT \| 13.3013728 \| 0.9993559 \| 0.9993735 \| \| AVPU \| 0.2135542 \| 1.0334403 \| 1.0201990 \| \| RPupilReact \| 9.4879059 \| 1.0446562 \| 1.0445606 \| \| RPupilSize \| 6.8642406 \| 1.2355947 \| 1.2227659 \| \| LPupilReact \| 9.1697538 \| 1.0489290 \| 1.0491825 \| \| LPupilSize \| 6.4937895 \| 1.2547893 \| 1.2512539 \|   **Table S3** Logistic Regression of continuous candidate predictor variables | | | | | | |  |
| **Variable** | **intercept** | | **ß** | **95% CI** | **p-value** | |  |
| Age | -5.2 | | 0.028 | 0.023 to 0.033 | | <0.001 |  |
| Resps | -5.9 | | 0.11 | 0.10 to 0.12 | | <0.001 |  |
| SpO2 | 6.6 | | -0.11 | -0.11 to -0.96 | | <0.001 |  |
| Pulse | -5.8 | | 0.026 | 0.023 to 0.028 | | <0.001 |  |
| SBP | -1.4 | | -0.015 | -0.018 to -0.011 | | <0.001 |  |
| DBP | -1.9 | | -0.018 | -0.023 to -0.013 | | <0.001 |  |
| Temp | -33.1 | | 0.8 | 0.72 to 0.88 | | <0.001 |  |
| BM | -3.6 | | 0.053 | 0.032 to 0.074 | | <0.001 |  |
| GCS_sum | -1.2 | | -0.15 | -0.18 to -0.12 | | <0.001 |  |
| RPupilSize | -2.9 | | -0.12 | -0.23 to -0.0089 | | 0.034 |  |
| ß – regression coefficient, CI – confidence interval | | | | | | |  |

| **Table S4** Logistic regression of categorical candidate predictor variables | | | | | |
| --- | --- | --- | --- | --- | --- |
| **Variable** | **Category** | **intercept** | **ß_i_** | **95% CI** | **p-value** |
| Imp | General medical | Reference category | | | |
|  | Other | -3.5 | -0.45 | -0.26 to 1.20 | 0.21 |
|  | Cardiovascular | -3.5 | -0.36 | -0.68 to -0.044 | 0.026 |
|  | Neurological | -3.5 | -1.80 | -2.50 to -1.20 | <0.001 |
|  | Obstetric/Gynae | -3.5 | -13.00 | -340 to 310 | 0.94 |
|  | Respiratory | -3.5 | 1.50 | 1.30 to 1.70 | <0.001 |
| Location | Home | Reference category | | | |
|  | Nursing home | -3.3 | 1.20 | 0.95 to 1.40 | <0.001 |
|  | Other | -3.3 | -0.94 | -1.20 to -0.65 | <0.001 |
| Gender | Male | Reference category | | | |
|  | Female | -3.2 | -0.026 | -0.19 to 0.14 | 0.75 |
| GCS_eye | Spontaneous | Reference category | | | |
|  | Verbal | -3.4 | 1.00 | 0.74 to 1.30 | <0.001 |
|  | Pain | -3.4 | 1.10 | 0.58 to 1.50 | <0.001 |
|  | No response | -3.4 | 1.10 | 0.60 to 1.60 | <0.001 |
| GCS_verbal | Oriented | Reference category | | | |
|  | Confused | -3.4 | 0.44 | 0.18 to 0.69 | <0.001 |
|  | Inappropriate words | -3.4 | 1.40 | 0.86 to 1.90 | <0.001 |
|  | Incomprehensible sounds | -3.4 | 1.30 | 0.87 to 1.70 | <0.001 |
|  | No response | -3.4 | 1.30 | 0.91 to 1.60 | <0.001 |
| GCS_motor | Obeys | Reference category | | | |
|  | Localises | -3.4 | 1.20 | 0.87 to 1.50 | <0.001 |
|  | Withdraws | -3.4 | 1.20 | 0.80 to 1.60 | <0.001 |
|  | Flexion | -3.4 | 0.83 | -0.61 to 2.30 | 0.26 |
|  | Extension | -3.4 | 2.30 | 0.98 to 3.60 | <0.001 |
|  | No response | -3.4 | 1.20 | 0.72 to 1.70 | <0.001 |
| Skin | Normal | Reference category | | | |
|  | Cyanosed | -3.7 | 1.80 | 1.40 to 2.30 | <0.001 |
|  | Flushed | -3.7 | 1.10 | 0.82 to 1.40 | <0.001 |
|  | Jaundice | -3.7 | 1.50 | 0.82 to 2.20 | <0.001 |
|  | Mottling | -3.7 | 2.20 | 1.40 to 2.90 | <0.001 |
|  | Pallor | -3.7 | 1.10 | 0.95 to 1.30 | <0.001 |
|  | Rash | -3.7 | -11.0 | -390 to 370 | 0.96 |
| CBRT | Normal | Reference category | | | |
|  | Delayed | -3.4 | 1.30 | 1.00 to 1.50 | <0.001 |
| AVPU | Alert | Reference category | | | |
|  | Verbal | -3.4 | 0.96 | 0.70 to 1.20 | 0.63 |
|  | Pain | -3.4 | 1.10 | 0.75 to 1.50 | 0.90 |
|  | No response | -3.4 | 0.99 | 0.49 to 1.50 | 0.001 |
| RPupilReact | Brisk | Reference category | | | |
|  | Sluggish | -3.3 | 0.55 | 0.26 to 0.84 | <0.001 |
|  | Fixed | -3.3 | -0.40 | -1.60 to 0.74 | 0.49 |
| ß – regression coefficient, CI – confidence interval | | | | | |

| **Table S5** Multivariable logistic regression model utilising GCS sum | | | | | | |
| --- | --- | --- | --- | --- | --- | --- |
| **Variable** |  | **ß_i_** | **lo 95** | **hi 95** | **p-value** | **Score assigned** |
|  | intercept | -5.46 | -5.98 | -4.93 | 0.00 |  |
| Location | home | reference | | | | 0 |
|  | nursing home | 0.21 | -0.14 | 0.56 | 0.23* | 0 |
|  | other | -0.28 | -0.64 | 0.08 | 0.13* | 0 |
| Age | below 40 | reference | | | | 0 |
|  | 40 to 49 | 0.12 | -0.58 | 0.81 | 0.74* | 0 |
|  | 50 to 59 | 0.40 | -0.20 | 1.00 | 0.19* | 0 |
|  | 60 to 69 | 0.82 | 0.30 | 1.33 | <0.001 | 1 |
|  | 70 to 79 | 0.94 | 0.45 | 1.43 | <0.001 | 1 |
|  | 80 to 89 | 0.87 | 0.37 | 1.36 | <0.001 | 1 |
|  | 90 to 99 | 1.35 | 0.79 | 1.92 | <0.001 | 1 |
|  | 100 plus | 1.59 | -0.17 | 3.35 | 0.08* | 0 |
| Respirations | below 10 | -13.20 | -948.66 | 922.25 | 0.98* | 0 |
|  | 10 to 20 | reference | | | | 0 |
|  | 21 to 25 | 0.52 | 0.19 | 0.86 | <0.001 | 1 |
|  | 26 to 30 | 0.89 | 0.56 | 1.22 | <0.001 | 1 |
|  | 31 to 35 | 1.00 | 0.57 | 1.44 | <0.001 | 1 |
|  | 36 to 40 | 1.28 | 0.91 | 1.65 | <0.001 | 1 |
|  | 41 to 45 | 2.14 | 1.46 | 2.81 | <0.001 | 2 |
|  | 46 to 50 | 0.87 | -0.03 | 1.76 | 0.06* | 0 |
|  | 51 to 55 | 1.27 | -0.17 | 2.70 | 0.08* | 0 |
|  | 56 to 60 | 1.96 | 1.02 | 2.91 | <0.001 | 2 |
|  | 60 plus | -12.60 | -3227.5 | 3202.2 | 0.99* | 0 |
| SpO2 | above 93 | reference | | | | 0 |
|  | 89 to 93 | 0.92 | 0.64 | 1.20 | <0.001 | 1 |
|  | 85 to 88 | 0.79 | 0.39 | 1.19 | <0.001 | 1 |
|  | below 85 | 1.19 | 0.84 | 1.55 | <0.001 | 1 |
| Pulse | below 60 | -0.58 | -1.43 | 0.27 | 0.18* | 0 |
|  | 60 to 100 | reference | | | | 0 |
|  | 101 to 110 | 0.62 | 0.29 | 0.94 | <0.001 | 1 |
|  | 111 to 120 | 0.63 | 0.29 | 0.97 | <0.001 | 1 |
|  | 121 to 130 | 0.96 | 0.58 | 1.34 | <0.001 | 1 |
|  | 131 to 140 | 0.88 | 0.42 | 1.33 | <0.001 | 1 |
|  | 141 to 150 | 1.84 | 1.34 | 2.35 | <0.001 | 2 |
|  | 151 to 160 | 1.11 | 0.39 | 1.83 | <0.001 | 1 |
|  | 161 to 170 | 0.38 | -0.86 | 1.63 | 0.55* | 0 |
|  | 171 to 180 | 0.04 | -2.11 | 2.18 | 0.97* | 0 |
|  | 180 plus | 1.07 | -0.13 | 2.28 | 0.08* | 0 |
| SBP | below 60 | 0.74 | -1.35 | 2.82 | 0.49* | 0 |
|  | 60 to 69 | 1.39 | 0.43 | 2.36 | <0.001 | 1 |
|  | 70 to 79 | 1.04 | 0.26 | 1.82 | 0.01 | 1 |
|  | 80 to 89 | 0.72 | 0.19 | 1.25 | 0.01 | 1 |
|  | 90 to 99 | 0.65 | 0.24 | 1.05 | <0.001 | 1 |
|  | 100 to 120 | reference | | | | 0 |
|  | 121 to 129 | -0.06 | -0.44 | 0.32 | 0.74* | 0 |
|  | 130 to 139 | -0.09 | -0.46 | 0.28 | 0.64* | 0 |
|  | 140 to 149 | -0.21 | -0.60 | 0.18 | 0.30* | 0 |
|  | 150 to 159 | -0.05 | -0.46 | 0.36 | 0.80* | 0 |
|  | 160 plus | -0.72 | -1.13 | -0.32 | <0.001 | -1 |
| GCS (sum) | 15 | reference | | | | 0 |
|  | 13 to 14 | -0.25 | -0.62 | 0.12 | 0.19* | 0 |
|  | 9 to 12 | 0.67 | 0.27 | 1.08 | <0.001 | 1 |
|  | 3 to 8 | 0.48 | -0.08 | 1.05 | 0.09* | 0 |
| Temperature | below 35.0 | -0.49 | -1.49 | 0.50 | 0.33* | 0 |
|  | 35.0 to 35.5 | 0.18 | -0.41 | 0.76 | 0.55* | 0 |
|  | 35.6 to 36.0 | -0.24 | -0.70 | 0.22 | 0.31* | 0 |
|  | 36.1 to 36.5 | -0.21 | -0.56 | 0.15 | 0.25* | 0 |
|  | 36.6 to 37.4 | reference | | | | 0 |
|  | 37.5 to 38.0 | 0.55 | 0.20 | 0.91 | <0.001 | 1 |
|  | 38.1 to 38.5 | 1.16 | 0.78 | 1.54 | <0.001 | 1 |
|  | 38.6 to 39.0 | 1.23 | 0.79 | 1.66 | <0.001 | 1 |
|  | 39.1 to 39.5 | 1.09 | 0.53 | 1.64 | <0.001 | 1 |
|  | 39.6 to 40.0 | 1.65 | 1.07 | 2.23 | <0.001 | 2 |
|  | above 40.0 | 1.91 | 1.09 | 2.74 | <0.001 | 2 |
| Skin | normal | reference | | | | 0 |
|  | cyanosed | -0.05 | -0.68 | 0.58 | 0.88* | 0 |
|  | flushed | 0.13 | -0.25 | 0.50 | 0.50* | 0 |
|  | jaundice | 0.90 | 0.01 | 1.79 | 0.05 | 1 |
|  | mottling | 0.82 | -0.33 | 1.96 | 0.16* | 0 |
|  | pallor | 0.40 | 0.14 | 0.66 | <0.001 | 0 |
|  | rash | -12.76 | -1121.5 | 1095.9 | 0.98* | 0 |
| CBRT | normal | reference | | | | 0 |
|  | delayed | -0.06 | -0.42 | 0.31 | 0.75* | 0 |

* Did not reach statistical significance

| **Table S6** Multivariable logistic regression model utilising GCS components | | | | | | | | |
| --- | --- | --- | --- | --- | --- | --- | --- | --- |
| **Variable** |  | **ß_i_** | | **lo 95** | **hi 95** | | **p-value** | **Score assigned** |
|  | Intercept | -5.46 | | -5.98 | -4.93 | | 0.00 |  |
| Location | home | reference | | | | | | 0 |
|  | nursing home | 0.20 | | -0.14 | 0.55 | | 0.25* | 0 |
|  | other | -0.28 | | -0.64 | 0.07 | | 0.12* | 0 |
| Age | under 40 | reference | | | | | | 0 |
|  | 40 to 49 | 0.10 | | -0.60 | 0.79 | | 0.79* | 0 |
|  | 50 to 59 | 0.39 | | -0.21 | 0.99 | | 0.20* | 0 |
|  | 60 to 69 | 0.79 | | 0.28 | 1.31 | | <0.001 | 1 |
|  | 70 to 79 | 0.93 | | 0.44 | 1.42 | | <0.001 | 1 |
|  | 80 to 89 | 0.84 | | 0.34 | 1.33 | | <0.001 | 1 |
|  | 90 to 99 | 1.33 | | 0.77 | 1.90 | | <0.001 | 1 |
|  | 100 plus | 1.63 | | -0.07 | 3.34 | | 0.06* | 0 |
| Respirations | below 10 | -13.47 | | -940.42 | 913.48 | | 0.98* | 0 |
|  | 10 to 20 | reference | | | | | | 0 |
|  | 21 to 25 | 0.53 | | 0.20 | | 0.87 | <0.001 | 1 |
|  | 26 to 30 | 0.89 | | 0.56 | | 1.22 | <0.001 | 1 |
|  | 31 to 35 | 1.01 | | 0.57 | | 1.45 | <0.001 | 1 |
|  | 36 to 40 | 1.30 | | 0.92 | | 1.67 | <0.001 | 1 |
|  | 41 to 45 | 2.12 | | 1.45 | | 2.80 | <0.001 | 2 |
|  | 46 to 50 | 0.85 | | -0.06 | | 1.75 | 0.07* | 0 |
|  | 51 to 55 | 1.21 | | -0.22 | | 2.64 | 0.10* | 0 |
|  | 56 to 60 | 1.94 | | 1.00 | | 2.88 | <0.001 | 2 |
|  | 60 plus | -12.59 | | -3230.28 | | 3205.09 | 0.99* | 0 |
| SpO2 | above 93 | reference | | | | | | 0 |
|  | 89 to 93 | 0.91 | | 0.63 | | 1.19 | <0.001 | 1 |
|  | 85 to 88 | 0.79 | | 0.38 | | 1.19 | <0.001 | 1 |
|  | below 85 | 1.18 | | 0.83 | | 1.53 | <0.001 | 1 |
| Pulse | below 60 | -0.58 | | -1.43 | 0.27 | | 0.18* | 0 |
|  | 60 to 100 | reference | | | | | | 0 |
|  | 101 to 110 | 0.64 | | 0.31 | 0.96 | | <0.001 | 1 |
|  | 111 to 120 | 0.63 | | 0.29 | 0.97 | | <0.001 | 1 |
|  | 21 to 130 | 0.96 | | 0.59 | 1.34 | | <0.001 | 1 |
|  | 131 to 140 | 0.87 | | 0.42 | 1.32 | | <0.001 | 1 |
|  | 141 to 150 | 1.84 | | 1.33 | 2.34 | | <0.001 | 2 |
|  | 151 to 160 | 1.09 | | 0.37 | 1.81 | | <0.001 | 1 |
|  | 161 to 170 | 0.45 | | -0.78 | 1.67 | | 0.47* | 0 |
|  | 171 to 180 | 0.01 | | -2.13 | 2.16 | | 0.99* | 0 |
|  | 180 plus | 1.05 | | -0.16 | 2.27 | | 0.09* | 0 |
| SBP | below 60 | 0.87 | | -1.11 | 2.85 | | 0.39* | 0 |
|  | 60 to 69 | 1.44 | | 0.48 | 2.40 | | <0.001 | 1 |
|  | 70 to 79 | 1.06 | | 0.29 | 1.83 | | 0.01 | 1 |
|  | 80 to 89 | 0.71 | | 0.18 | 1.25 | | 0.01 | 1 |
|  | 90 to 99 | 0.64 | | 0.24 | 1.05 | | <0.001 | 1 |
|  | 100 to 120 | reference | | | | | | 0 |
|  | 121 to 129 | -0.07 | | -0.45 | 0.31 | | 0.71* | 0 |
|  | 130 to 139 | -0.08 | | -0.46 | 0.29 | | 0.66* | 0 |
|  | 140 to 149 | -0.19 | | -0.59 | 0.20 | | 0.33* | 0 |
|  | 150 to 159 | -0.07 | | -0.48 | 0.34 | | 0.75* | 0 |
|  | 160 plus | -0.72 | | -1.13 | -0.32 | | <0.001 | -1 |
| GCS (verbal) | oriented | reference | | | | | | 0 |
|  | confused | | 0.02 | -0.33 | 0.36 | | 0.92* | 0 |
|  | Inapp. words | | 0.50 | -0.25 | 1.25 | | 0.19* | 0 |
|  | Incomp. sounds | | 0.46 | -0.21 | 1.13 | | 0.18* | 0 |
|  | no response | 0.83 | | 0.31 | 1.34 | | <0.001 | 1 |
| Temperature | below 35 | -0.50 | | -1.49 | 0.48 | | 0.32* | 0 |
|  | 35.0 to 35.5 | 0.17 | | -0.42 | 0.75 | | 0.58* | 0 |
|  | 35.6 to 36.0 | -0.25 | | -0.71 | 0.21 | | 0.28* | 0 |
|  | 36.1 to 36.5 | -0.21 | | -0.56 | 0.15 | | 0.25* | 0 |
|  | 36.6 to 37.4 | reference | | | | | | 0 |
|  | 37.5 to 38.0 | 0.55 | | 0.19 | | 0.90 | <0.001 | 1 |
|  | 38.1 to 38.5 | 1.15 | | 0.78 | | 1.53 | <0.001 | 1 |
|  | 38.6 to 39.0 | 1.22 | | 0.78 | | 1.66 | <0.001 | 1 |
|  | 39.1 to 39.5 | 1.09 | | 0.53 | | 1.64 | <0.001 | 1 |
|  | 39.6 to 40.0 | 1.62 | | 1.04 | | 2.20 | <0.001 | 2 |
|  | above 40 | 1.89 | | 1.06 | | 2.72 | <0.001 | 2 |
| Skin | normal | reference | | | | | | 0 |
|  | cyanosed | 0.00 | | -0.63 | | 0.62 | 1.00* | 0 |
|  | flushed | 0.13 | | -0.24 | | 0.51 | 0.48* | 0 |
|  | jaundice | 0.89 | | 0.00 | | 1.78 | 0.05 | 1 |
|  | mottling | 0.79 | | -0.36 | | 1.93 | 0.18* | 0 |
|  | pallor | 0.40 | | 0.14 | | 0.66 | <0.001 | 0 |
|  | rash | -12.76 | | -1121.39 | | 1095.86 | 0.98* | 0 |
| CBRT | normal | Reference | | | | | | 0 |
|  | delayed | -0.06 | | -0.42 | 0.31 | | 0.76* | 0 |

* Did not reach statistical significance

| **Table S7** Multivariable logistic regression model utilising AVPU | | | | | | |
| --- | --- | --- | --- | --- | --- | --- |
| **Variable** |  | **ß_i_** | **lo 95** | **hi 95** | **p-value** | **Score assigned** |
|  | intercept | -5.50 | -6.02 | -4.97 | 0.00 |  |
| Location | home | reference | | | | 0 |
|  | nursing home | 0.24 | -0.09 | 0.58 | 0.15* | 0 |
|  | other | -0.28 | -0.64 | 0.07 | 0.12* | 0 |
| Age | below 40 | reference | | | | 0 |
|  | 40 to 49 | 0.14 | -0.55 | 0.84 | 0.69* | 0 |
|  | 50 to 59 | 0.45 | -0.16 | 1.05 | 0.15* | 0 |
|  | 60 to 69 | 0.83 | 0.32 | 1.34 | <0.001 | 1 |
|  | 70 to 79 | 0.98 | 0.49 | 1.47 | <0.001 | 1 |
|  | 80 to 89 | 0.88 | 0.38 | 1.37 | <0.001 | 1 |
|  | 90 to 99 | 1.37 | 0.81 | 1.93 | <0.001 | 1 |
|  | 100 plus | 1.34 | -0.40 | 3.08 | 0.13* | 0 |
| Respirations | below 10 | -13.25 | -945.34 | 918.84 | 0.98* | 0 |
|  | 10 to 20 | reference | | | | 0 |
|  | 21 to 25 | 0.54 | 0.21 | 0.87 | <0.001 | 1 |
|  | 26 to 30 | 0.91 | 0.58 | 1.24 | <0.001 | 1 |
|  | 31 to 35 | 1.01 | 0.57 | 1.45 | <0.001 | 1 |
|  | 36 to 40 | 1.32 | 0.95 | 1.69 | <0.001 | 1 |
|  | 41 to 45 | 2.15 | 1.49 | 2.80 | <0.001 | 2 |
|  | 46 to 50 | 0.80 | -0.10 | 1.69 | 0.08* | 0 |
|  | 51 to 55 | 1.23 | -0.18 | 2.64 | 0.09* | 0 |
|  | 56 to 60 | 1.99 | 1.05 | 2.93 | <0.001 | 2 |
|  | 60 plus | -12.55 | -3229.45 | 3204.36 | 0.99* | 0 |
| SpO2 | above 93 | reference | | | | 0 |
|  | 89 to 93 | 0.90 | 0.62 | 1.18 | <0.001 | 1 |
|  | 85 to 88 | 0.76 | 0.35 | 1.16 | <0.001 | 1 |
|  | below 85 | 1.16 | 0.81 | 1.50 | <0.001 | 1 |
| Pulse | below 60 | -0.58 | -1.42 | 0.27 | 0.18* | 0 |
|  | 60 to 100 | reference | | | | 0 |
|  | 101 to 110 | 0.62 | 0.30 | 0.95 | <0.001 | 1 |
|  | 111 to 120 | 0.64 | 0.30 | 0.98 | <0.001 | 1 |
|  | 121 to 130 | 0.99 | 0.61 | 1.36 | <0.001 | 1 |
|  | 131 to 140 | 0.89 | 0.45 | 1.33 | <0.001 | 1 |
|  | 141 to 150 | 1.88 | 1.38 | 2.37 | <0.001 | 2 |
|  | 151 to 160 | 1.14 | 0.43 | 1.85 | <0.001 | 1 |
|  | 161 to 170 | 0.74 | -0.39 | 1.87 | 0.20* | 0 |
|  | 171 to 180 | 0.07 | -2.07 | 2.21 | 0.95* | 0 |
|  | 180 plus | 1.12 | -0.08 | 2.33 | 0.07* | 0 |
| SBP | below 60 | 0.72 | -1.33 | 2.78 | 0.49* | 0 |
|  | 60 to 69 | 1.40 | 0.45 | 2.36 | <0.001 | 1 |
|  | 70 to 79 | 1.01 | 0.23 | 1.79 | 0.01 | 1 |
|  | 80 to 89 | 0.73 | 0.20 | 1.26 | 0.01 | 1 |
|  | 90 to 99 | 0.64 | 0.24 | 1.04 | <0.001 | 1 |
|  | 100 to 120 | reference | | | | 0 |
|  | 121 to 129 | -0.10 | -0.47 | 0.28 | 0.62* | 0 |
|  | 130 to 139 | -0.11 | -0.48 | 0.27 | 0.58* | 0 |
|  | 140 to 149 | -0.20 | -0.59 | 0.19 | 0.30* | 0 |
|  | 150 to 159 | -0.08 | -0.49 | 0.32 | 0.69* | 0 |
|  | 160 plus | -0.71 | -1.11 | -0.31 | <0.001 | -1 |
| AVPU | alert | reference | | | | 0 |
|  | verbal | 0.44 | 0.08 | 0.80 | 0.02 | 0 |
|  | pain | 0.48 | -0.12 | 1.07 | 0.12* | 0 |
|  | no response | 0.61 | -0.13 | 1.36 | 0.11* | 0 |
| Temperature | below 35.0 | -0.50 | -1.49 | 0.49 | 0.32* | 0 |
|  | 35.0 to 35.5 | 0.13 | -0.45 | 0.71 | 0.66* | 0 |
|  | 35.6 to 36.0 | -0.28 | -0.74 | 0.18 | 0.23* | 0 |
|  | 36.1 to 36.5 | -0.19 | -0.54 | 0.16 | 0.28* | 0 |
|  | 36.6 to 37.4 | reference | | | | 0 |
|  | 37.5 to 38.0 | 0.53 | 0.17 | 0.88 | <0.001 | 1 |
|  | 38.1 to 38.5 | 1.17 | 0.79 | 1.54 | <0.001 | 1 |
|  | 38.6 to 39.0 | 1.17 | 0.74 | 1.60 | <0.001 | 1 |
|  | 39.1 to 39.5 | 1.13 | 0.59 | 1.67 | <0.001 | 1 |
|  | 39.6 to 40.0 | 1.60 | 1.02 | 2.18 | <0.001 | 2 |
|  | above 40.0 | 1.90 | 1.07 | 2.73 | <0.001 | 2 |
| Skin | normal | reference | | | | 0 |
|  | cyanosed | -0.10 | -0.73 | 0.53 | 0.76* | 0 |
|  | flushed | 0.12 | -0.26 | 0.49 | 0.54* | 0 |
|  | jaundice | 0.82 | -0.08 | 1.71 | 0.07* | 0 |
|  | mottling | 0.99 | -0.06 | 2.05 | 0.07* | 0 |
|  | pallor | 0.39 | 0.13 | 0.65 | <0.001 | 0 |
|  | rash | -12.73 | -1122.95 | 1097.49 | 0.98* | 0 |
| CBRT | normal | Reference | | | | 0 |
|  | delayed | -0.04 | -0.40 | 0.32 | 0.82* | 0 |

* Did not reach statistical significance

| **Table S8** Final weighted scores for candidate model using GCS sum | | |
| --- | --- | --- |
| **Variable** | **Interval** | **Score** |
| Age | Under 60 | 0 |
|  | 60 or older | 1 |
| Respirations | 20 or lower | 0 |
|  | 21 to 40 | 1 |
|  | 41 to 60 | 2 |
|  | 61 or over | 0 |
| SpO2 | 94 or higher | 0 |
|  | 93 or lower | 1 |
| Pulse | 100 or lower | 0 |
|  | 101 to 140 | 1 |
|  | 141 to 160 | 2 |
|  | 161 or over | 0 |
| SBP | Under 60 | 0 |
|  | 60 to 99 | 1 |
|  | 100 to 159 | 0 |
|  | 160 or higher | -1 |
| Temperature | 37.4 or lower | 0 |
|  | 37.5 to 39.5 | 1 |
|  | 39.6 or higher | 2 |
| Skin | Jaundice, pallor, mottling | 1 |
|  | Any other | 0 |
| GCS (sum) | 13 to 15 | 0 |
|  | 3 to 12 | 1 |

| **Table S9** Final weighted scores for candidate model using GCS components | | |
| --- | --- | --- |
| **Variable** | **Interval** | **Score** |
| Age | Under 60 | 0 |
|  | 60 or older | 1 |
| Respirations | 20 or lower | 0 |
|  | 21 to 40 | 1 |
|  | 41 to 60 | 2 |
|  | 61 or over | 0 |
| SpO2 | 94 or higher | 0 |
|  | 93 or lower | 1 |
| Pulse | 100 or lower | 0 |
|  | 101 to 140 | 1 |
|  | 141 to 160 | 2 |
|  | 161 or over | 0 |
| SBP | Under 60 | 0 |
|  | 60 to 99 | 1 |
|  | 100 to 159 | 0 |
|  | 160 or higher | -1 |
| Temperature | 37.4 or lower | 0 |
|  | 37.5 to 39.5 | 1 |
|  | 39.6 or higher | 2 |
| Skin | Jaundice, pallor, mottling | 1 |
|  | Any other | 0 |
| GCS (verbal) | Any response | 0 |
|  | No response | 1 |

| **Table S10** Final weighted scores for candidate model using AVPU | | |
| --- | --- | --- |
| **Variable** | **Interval** | **Score** |
| Age | Under 60 | 0 |
|  | 60 or older | 1 |
| Respirations | 20 or lower | 0 |
|  | 21 to 40 | 1 |
|  | 41 to 60 | 2 |
|  | 61 or over | 0 |
| SpO2 | 94 or higher | 0 |
|  | 93 or lower | 1 |
| Pulse | 100 or lower | 0 |
|  | 101 to 140 | 1 |
|  | 141 to 160 | 2 |
|  | 161 or over | 0 |
| SBP | Under 60 | 0 |
|  | 60 to 99 | 1 |
|  | 100 to 159 | 0 |
|  | 160 or higher | -1 |
| Temperature | 37.4 or lower | 0 |
|  | 37.5 to 39.5 | 1 |
|  | 39.6 or higher | 2 |

| **Table S11** Operating characteristics for the SEPSIS score | | | | | | | | | |
| --- | --- | --- | --- | --- | --- | --- | --- | --- | --- |
| **Score** | ≥1 | ≥2 | ≥3 | ≥4 | ≥5 | ≥6 | ≥7 | ≥8 | ≥9 |
| **Patients with undifferentiated medical complaints** | | | |  |  |  |  |  |  |
| Sens | 0.99 (0.98-1.00) | 0.94 (0.92-0.96) | 0.82 (0.79-0.85) | 0.63 (0.59-0.67) | 0.39 (0.35-0.43) | 0.22 (0.18-0.25) | 0.09 (0.11-0.17) | 0.03(0.02-0.04) | 0.01 (0.0-0.02) |
| Spec | 0.24 (0.23-0.25) | 0.55 (0.55-0.56) | 0.77 (0.76-0.78) | 0.89 (0.89-0.90) | 0.96 (0.96-0.96) | 0.99 (0.99-0.99) | 1.0 (1.0-1.0) | 1.0 (1.0-1.0) | 1.0 (1.0-1.0) |
| PPV | 0.05 (0.04-0.05) | 0.07 (0.07-0.08) | 0.12 (0.11-0.13) | 0.19 (0.17-0.20) | 0.27 (0.24-0.30) | 0.40 (0.34-0.45) | 0.51 (0.41-0.61) | 0.57 (0.37-0.76) | 0.80 (0.28-0.99) |
| NPV | 1.00(1.00-1.00) | 1.00(0.99-1.00) | 0.99 (0.99-1.00) | 0.98 (0.98-0.99) | 0.98 (0.97-0.98) | 0.97 (0.97-0.97) | 0.97 (0.96-0.97) | 0.96 (0.96-0.97) | 0.96 (0.96-0.97) |
| PLR | 1.30 (1.29-1.32) | 2.11 (2.06-2.17) | 3.57 (3.41-3.75) | 5.96 (5.52-6.43) | 9.48 (8.35-10.76) | 17.0 (13.9-21.0) | 27.62 (19.01-40.1) | 34.8 (16.5-73.2) | 104.4 (11.7-932.2) |
| NLR | 0.04 (0.01-0.08) | 0.10 (0.07-0.14) | 0.23 (0.19-0.27) | 0.41 (0.37-0.46) | 0.64 (0.60-0.68) | 0.79 (0.76-0.83) | 0.91 (0.89-0.94) | 0.97 (0.96-0.99) | 0.99 (0.98-1.0) |
| **Patients with infection** | | | | | | | | | |
| Sens | 0.99 (0.98-1.00) | 0.94 (0.92-0.96) | 0.82 (0.79-0.85) | 0.63 (0.59-0.67) | 0.39 (0.35-0.43) | 0.22 (0.18-0.25) | 0.09 (0.11-0.17) | 0.03 (0.02-0.04) | 0.01 (0.0-0.02) |
| Spec | 0.86 (0.86-0.87) | 0.90 (0.92-0.96) | 0.94 (0.93-0.94) | 0.97 (0.96-0.97) | 0.99 (0.98-0.99) | 0.99 (0.99-1.0) | 1.0 (1.0-1.0) | 1.0 (1.0-1.0) | 1.0 (1.0-1.0) |
| PPV | 0.21 (0.20-0.23) | 0.26 (0.24-0.28) | 0.33 (0.31-0.36) | 0.41 (0.38-0.44) | 0.5 (0.45-0.55) | 0.62 (0.55-0.68) | 0.71 (0.60-0.81) | 0.76 (0.53-0.92) | 0.80 (0.28-0.99) |
| NPV | 1.0 (1.0-1.0) | 1.0 (1.0-1.0) | 0.99 (0.99-0.99) | 0.99 (0.98-0.99) | 0.98 (0.97-0.98) | 0.97 (0.97-0.97) | 0.97 (0.96-0.97) | 0.96 (0.96-0.97) | 0.96 (0.96-0.97) |
| PLR | 7.14 (6.86-7.43) | 9.33 (8.87-9.82) | 13.0 (12.1-13.9) | 18.1 (16.4-20.1) | 26.1 (22.2-30.1) | 41.7 (32.0-54.5) | 64.0 (39.3-104.4) | 83.5 (30.7-227.1) | 104.3 (11.7-932.2) |
| NLR | 0.01 (0.00-0.02) | 0.06 (0.05-0.09) | 0.19 (0.16-0.22) | 0.38 (0.34-0.42) | 0.62 (0.58-0.66) | 0.79 (0.76-0.82) | 0.91 (0.89-0.93) | 0.97 (0.96-0.99) | 0.99 (0.98-1.0) |
| Sens-sensitivity, Spec-specificity, PPV-positive predictive value, NPV-negative predictive value, PLR-positive likelihood ratio, NLR-negative likelihood ratio, brackets () 95% confidence interval | | | | | | | | | |

Figure S1 Convergence plots


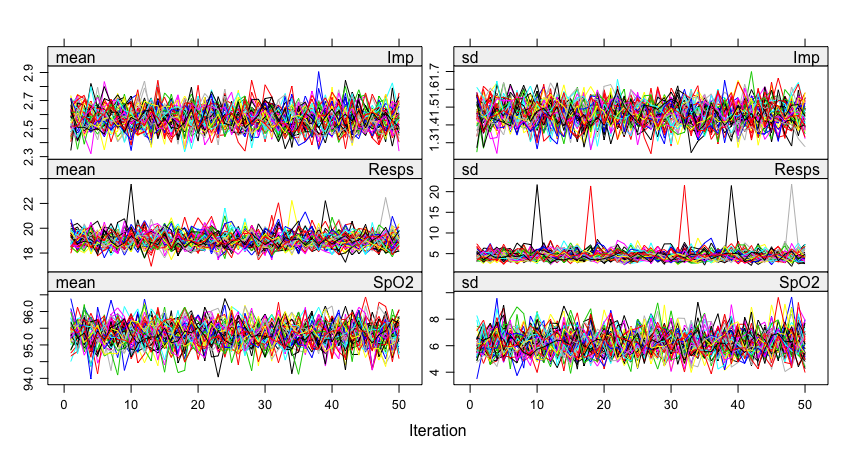


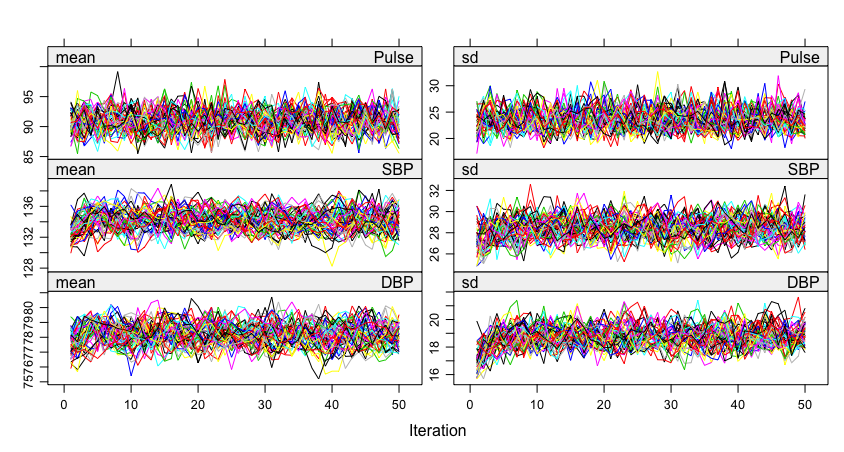


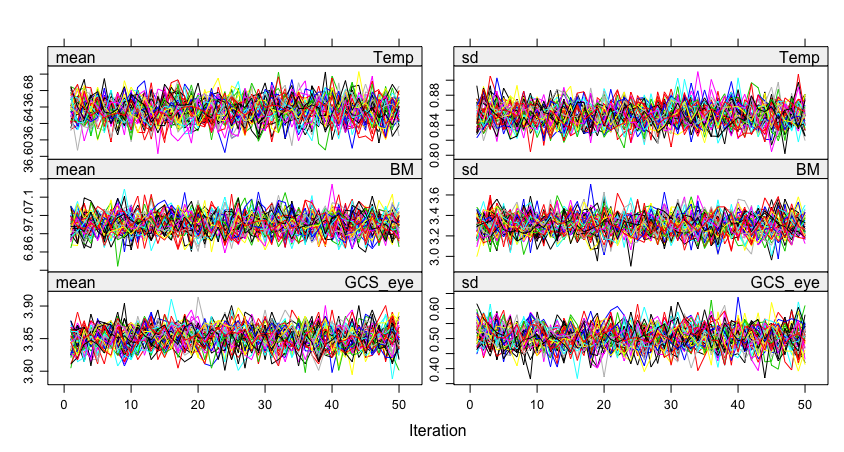


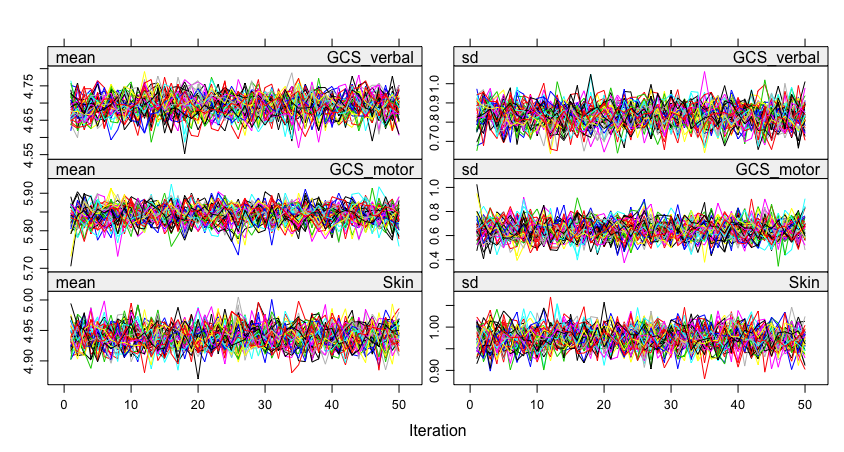


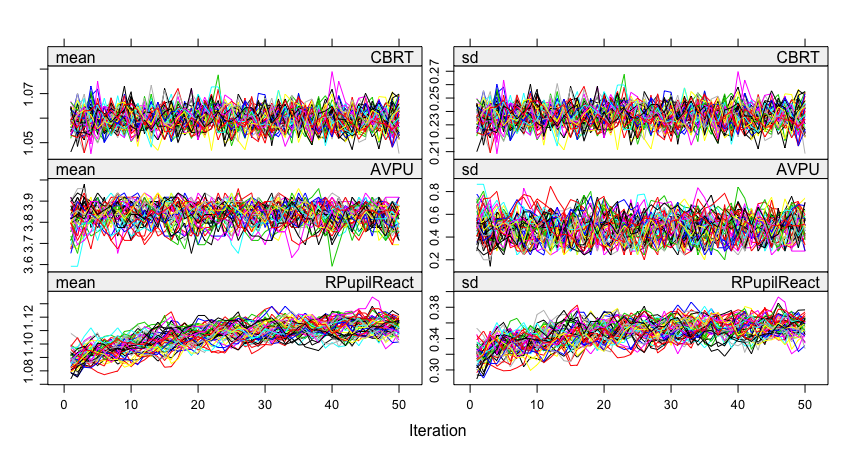


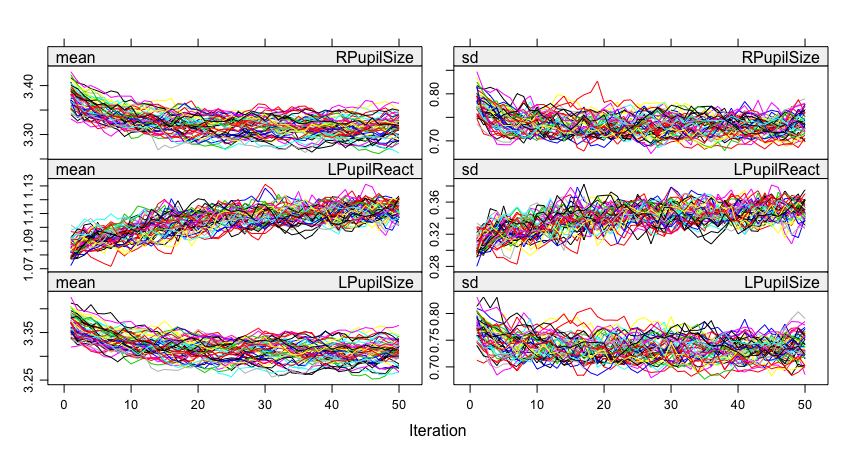


**Figure S2** Density plots

Density plot Resps


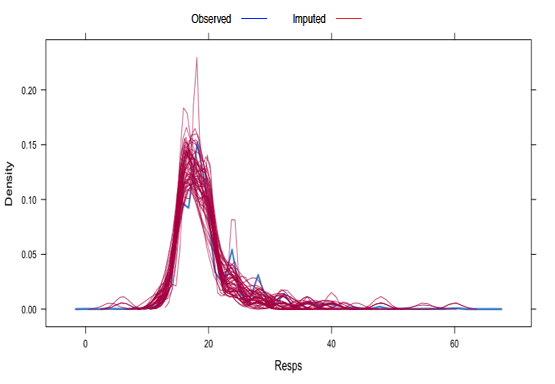


Density plot SpO_2_


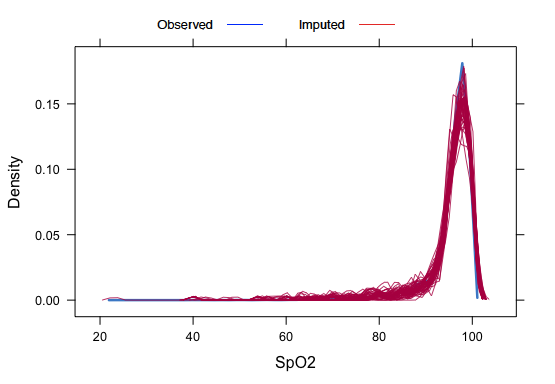


Density plot Pulse


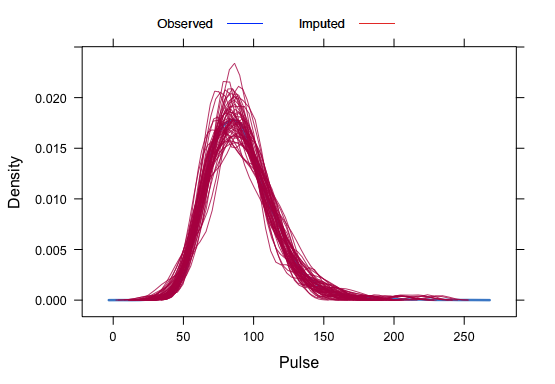


Density plot SBP


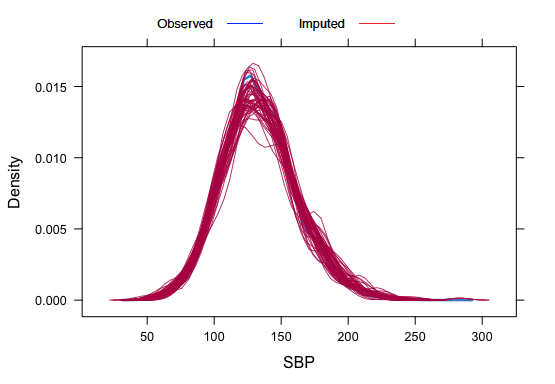


Density plot DBP


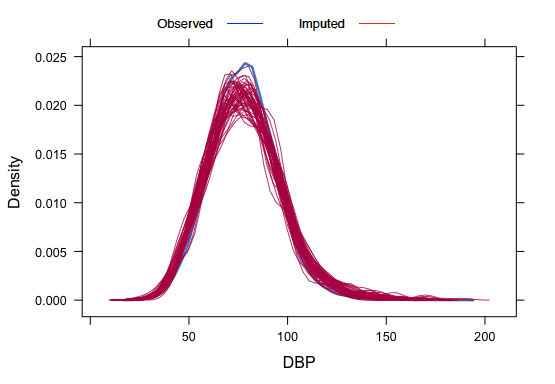


Density plot BM


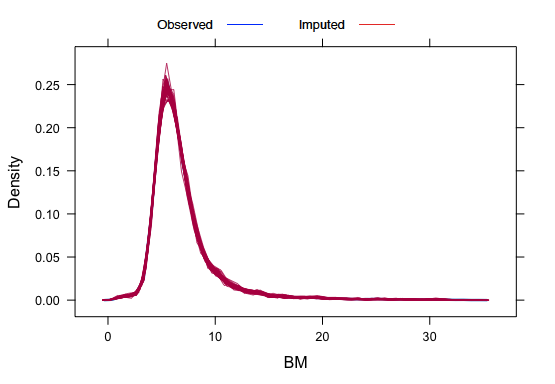


Density plot Temp


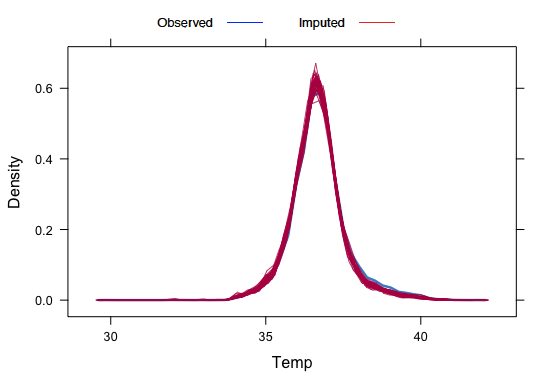


Density plot RPupilSize


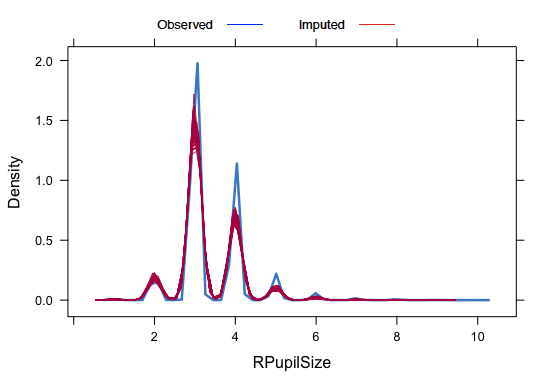


**Figure S3** Box and whisker plots


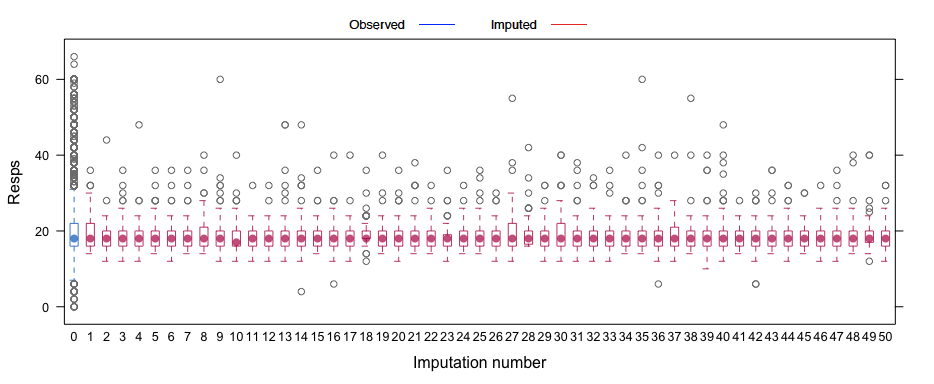
Box and Whisker plot Resps


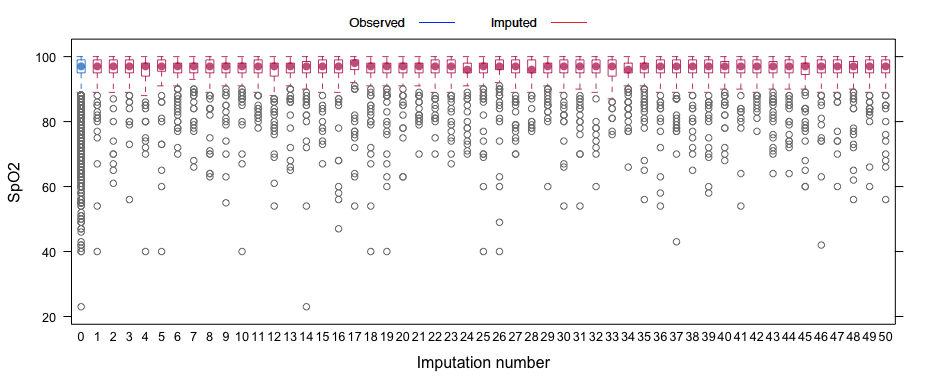
Box and Whisker plot SpO2


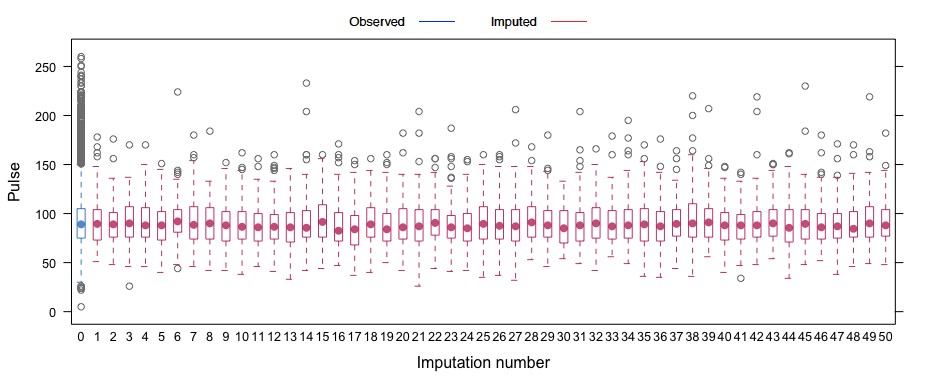
Box and Whisker plot Pulse


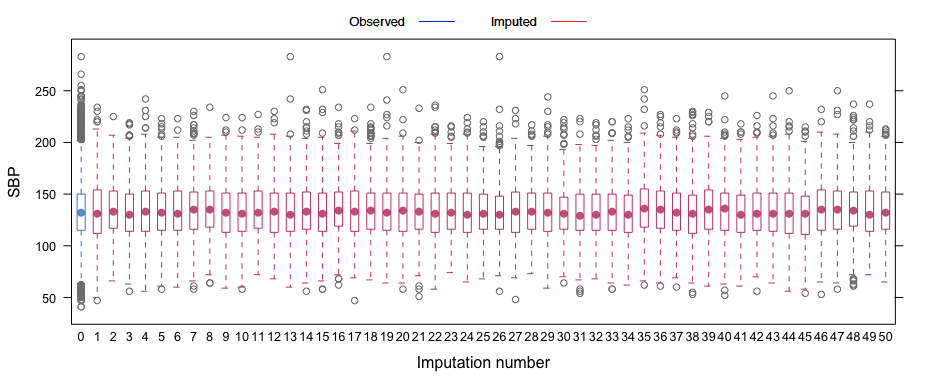
Box and Whisker plot SBP


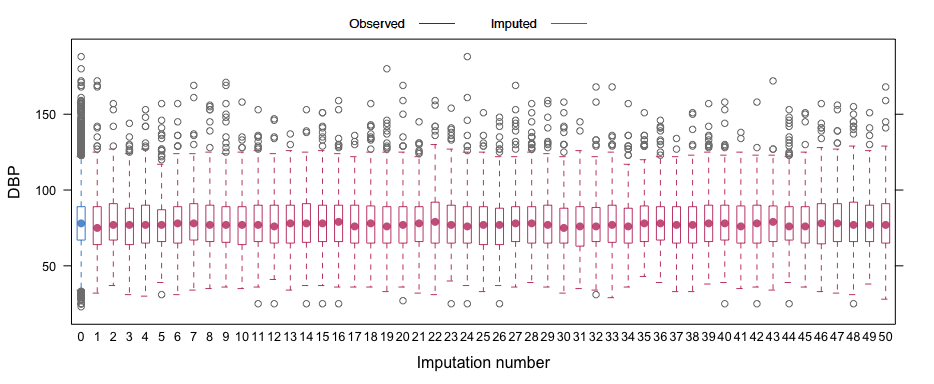
Box and Whisker plot DBP

Box and Whisker plot BM


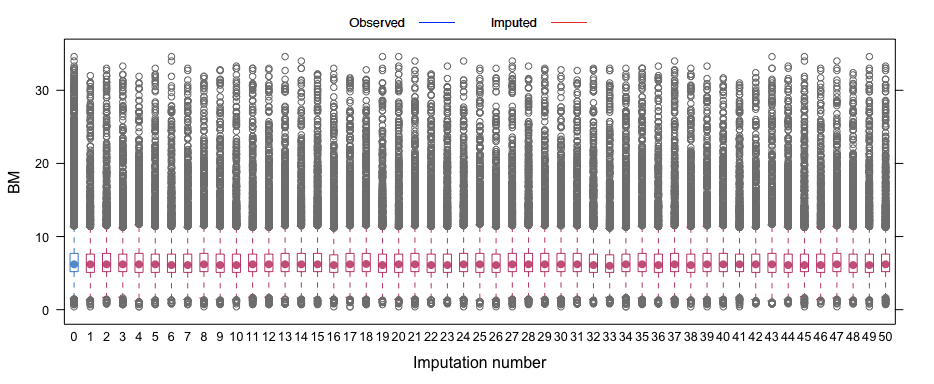


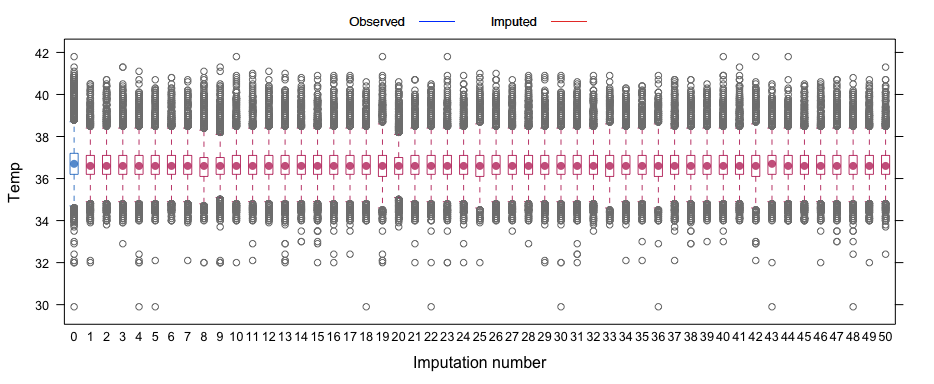
Box and Whisker plot Temp

Box and Whisker plot RPupilSize


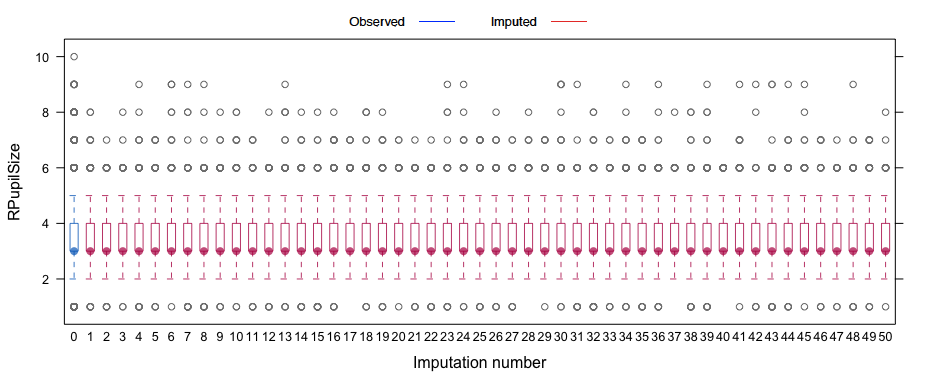


Figure S4 Colinearity between candidate predictor variables


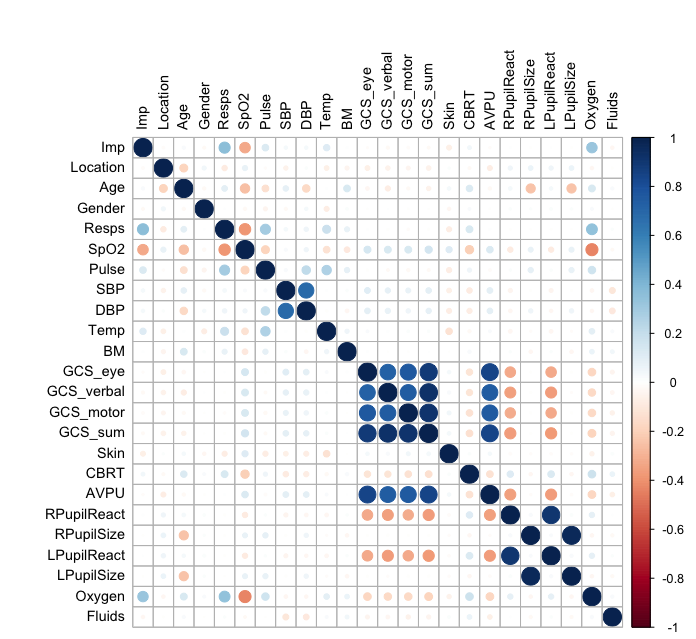


Blue circles indicate a positive relationship, while red circles indicate an inverse relationship. Darker colour shades and increasing circle size indicate increasing strength of the relationship. The dark blue diagonal represents the perfect relationship of each variable with itself.
